# Role of hydraulic traits in stomatal regulation of transpiration under different vapour pressure deficits across five Mediterranean tree crops

**DOI:** 10.1093/jxb/erad157

**Published:** 2023-04-28

**Authors:** Virginia Hernandez-Santana, Celia M Rodriguez-Dominguez, Jaime Sebastian-Azcona, Luis Felipe Perez-Romero, Antonio Diaz-Espejo

**Affiliations:** Irrigation and Ecophysiology Group. Instituto de Recursos Naturales y Agrobiología (IRNAS), Consejo Superior de Investigaciones Científicas (CSIC), Avda Reina Mercedes, 41012 Seville, Spain; Laboratory of Plant Molecular Ecophysiology, Instituto de Recursos Naturales y Agrobiología (IRNAS), Consejo Superior de Investigaciones Científicas (CSIC), Avda Reina Mercedes, 41012 Seville, Spain; Irrigation and Ecophysiology Group. Instituto de Recursos Naturales y Agrobiología (IRNAS), Consejo Superior de Investigaciones Científicas (CSIC), Avda Reina Mercedes, 41012 Seville, Spain; Laboratory of Plant Molecular Ecophysiology, Instituto de Recursos Naturales y Agrobiología (IRNAS), Consejo Superior de Investigaciones Científicas (CSIC), Avda Reina Mercedes, 41012 Seville, Spain; Irrigation and Ecophysiology Group. Instituto de Recursos Naturales y Agrobiología (IRNAS), Consejo Superior de Investigaciones Científicas (CSIC), Avda Reina Mercedes, 41012 Seville, Spain; Escuela Técnica Superior de Ingeniería, Universidad de Huelva, Avenida del Ejercito s/n. 21007 Huelva, Spain; Irrigation and Ecophysiology Group. Instituto de Recursos Naturales y Agrobiología (IRNAS), Consejo Superior de Investigaciones Científicas (CSIC), Avda Reina Mercedes, 41012 Seville, Spain; Laboratory of Plant Molecular Ecophysiology, Instituto de Recursos Naturales y Agrobiología (IRNAS), Consejo Superior de Investigaciones Científicas (CSIC), Avda Reina Mercedes, 41012 Seville, Spain; University of Essex, UK

**Keywords:** Almond, grapefruit, Huber value, lemon, olive, orange, plant hydraulic conductance, sap flux density, stomatal conductance, vapour pressure deficit

## Abstract

The differential stomatal regulation of transpiration among plant species in response to water deficit is not fully understood, although several hydraulic traits have been reported to influence it. This knowledge gap is partly due to a lack of direct and concomitant experimental data on transpiration, stomatal conductance, and hydraulic traits. We measured sap flux density (*J*_s_), stomatal conductance (*g*_s_), and different hydraulic traits in five crop species. Our aim was to contribute to establishing the causal relationship between water consumption and its regulation using a hydraulic trait-based approach. The results showed that the species-specific regulation of *J*_s_ by *g*_s_ was overall coordinated with the functional hydraulic traits analysed. Particularly relevant was the negative and significant relationship found between the Huber value (*H*_v_) and its functional analogue ratio between maximum *J*_s_ and *g*_s_ (*J*_smax_/*g*_smax_) which can be understood as a compensation to maintain the hydraulic supply to the leaves. The *H*_v_ was also significantly related to the slope of the relationship between *g*_s_ and *J*_s_ response to vapour pressure deficit and explained most of its variability, adding up to evidence recognizing *H*_v_ as a major trait in plant water relations. Thus, a hydraulic basis for regulation of tree water use should be considered.

## Teaser

We found coordination between stomatal regulation of tree transpiration and hydraulic traits, with the Huber value being the trait with the most predominant role in the stomatal control–water use relationship.

## Introduction

Transpiration (*E*) is a large component of the water cycle in natural systems, accounting for 39% of terrestrial precipitation and 61% of evapotranspiration globally (Schlesinger and Jasechko, 2014). Water loss from plants is mainly controlled by stomata that respond greatly to soil water deficit; however, under non-limiting conditions, *E* is basically a function of air vapour pressure deficit (*D*) and radiation (Penman, 1948; Monteith, 1965). *D* is representative of the degree of atmospheric drought and the main determinant of stomatal control of *E* in tightly coupled canopies, whereas, in uncoupled canopies, radiation acts as the main variable, *E* being mainly limited by boundary layer or aerodynamic conductance, depending on the scale considered (Jarvis and McNaughton, 1986). The plant characteristics that differentiate the species sensitivity of transpiration regulation by stomata in response to water stress, produced either by soil or by atmosphere drought, have received much less attention than the general mechanisms acting on faster time scales on stomatal conductance (*g*_s_) control (see, for example, Buckley, 2019 for a comprehensive review on the latest major advances in stomatal water relations).

Among the species-specific characteristics that produce differential *g*_s_ regulation, hydraulic traits would be key, as there is sufficient evidence that these traits are closely coordinated with gas exchange (e.g. Sperry *et al.*, 2016; Meinzer, 2002; Maherali *et al.*, 2006; Brodribb *et al.*, 2010; Klein, 2014; Hernandez-Santana *et al.*, 2016; Scoffoni *et al.*, 2016). The coordination of *g*_s_ with hydraulic traits seems to indicate a stomatal regulation to prevent cavitation episodes from occurring within the plant vascular system (Tyree and Zimmermann, 2002). Despite this ample evidence on the gas exchange–plant hydraulic traits relationship, our understanding of the interspecific variation of this relationship with the regulation of transpiration is more limited. Indeed, direct experimental support on the comparison between stomatal and plant hydraulic responses to declining tree water use measurements has rarely been reported, mainly due to the limitation of available data including all these variables (Flo *et al.*, 2021). The current extended use of methods to estimate sap flow (Poyatos *et al.*, 2021), which allows the calculation of tree transpiration, could improve this limited data availability issue. Leaf hydraulic conductance (Sack and Holbrook, 2006) and allocation ratios between plant tissues/organs involved in water loss, transport, and water uptake, such as the Huber value (*H*_v_) (Magnani *et al.*, 2002; Addington *et al.*, 2006; Martínez-Vilalta *et al.*, 2009; Martin-StPaul *et al.*, 2013; Hernandez-Santana *et al.*, 2019), among other hydraulic traits, influence the efficiency with which transpired water is replaced, and thus the degree to which stomata can remain open. Specifically, *H*_v_ is central to plant water balance and drought response, but its coordination with other ecophysiological traits is poorly understood, which hinders the development of trait-based prediction models. Therefore, besides helping to establish the causal relationship between *E* and its regulation by *g*_s_ (Litvak *et al.*, 2012; Du *et al.*, 2020), species-specific hydraulic traits could be crucial for models predicting climate change impacts on ecosystems, agricultural yields, and biosphere–atmosphere feedbacks (Anderegg, 2020). In most global vegetation and ecosystem models, plant water limitation is represented with a rarely tested empirical function, and thus the vegetation models present a poor performance in calculating carbon and water budgets during drought conditions and a large degree of uncertainty in future predictions (Hanson *et al.*, 2004; Medlyn *et al.*, 2015; Ukkola *et al.*, 2016; Trugman *et al.*, 2018). The incorporation of hydraulic traits in coordination with *g*_s_ in vegetation models has a great potential for improved and more mechanistic simulation and prediction of water stress responses at the plant, ecosystem, and global scale to a changing climate on a long time scale (Novick *et al.*, 2009; Grossiord *et al.*, 2020; Flo *et al.*, 2021). This approach includes the response to both soil water deficit and increasing *D* which, despite the unprecedented increment expected to occur due to climate change (Grossiord *et al.*, 2020), is not included in these models (Trugman *et al.*, 2018). Moreover, integrating hydraulic traits would contribute to including a differential sensitivity of plant species to reduced water availability, which has not been considered yet (Sabot *et al.*, 2020). In addition, the joint study of hydraulic traits and plant *E* could help to understand how anatomical hydraulic traits of different organs are coordinated with plant *E*, which is still not fully understood (McCulloh *et al.*, 2019).

In this work, we analyse the role of hydraulic properties across five fruit tree species of high agricultural interest (almond, olive, lemon, grapefruit, and orange) in the regulation of sap flux density (*J*_s_) by *g*_s_ as atmospheric drought increases (i.e. *D* increases). The analyses are separated into two subsequent specific objectives: (i) to analyse the coordination between *J*_s_ limitation response to increasing *D* with species-specific hydraulic traits, determined both anatomically and functionally; and (ii) to merge the results obtained by species in the previous analyses into a generalized behavioural model. Our main hypothesis is that any deviations from the linear relationship between *J*_s_ and *D* would be indicative of stomatal regulatory responses in the studied species, and we further hypothesize that this *J*_s_ limitation would be species specific and coordinated with their hydraulic properties. We also expect to find a relationship between anatomical hydraulic properties and their function. Finally, we hypothesize that *g*_s_ can be explained through *J*_s_ and the hydraulic trait *H*_v_.

## Materials and methods

### Field site

The study was conducted in the Research Farm La Hampa-CSIC located in Seville (Spain, 37°17ʹN, –6°3ʹW) from May to October of 2020. The study was conducted in 5-year-old trees of five species (almond, *Prunus dulcis*; grapefruit, *Citrus×paradisi*; lemon, *Citrus×limon*; olive, *Olea europaea*; and orange, *Citrus sinensis*), planted in 2018, in a 6 m×4 m formation. The trees were distributed in two plots of 480 m^2^ per species, with 20 trees per plot. Four central trees were used for most of the measurements. The soil in the Research Farm is a sandy loam Xerochrept (USDA 2010), with a depth of 0.9–2 m.

The climate of the area is Mediterranean, with mild, rainy winters and hot, dry summers, with hardly any rain during the months of the study. Average annual potential evapotranspiration (*ET*_o_) and precipitation are 1176 mm and 470 mm, respectively (La Hampa-CSIC). Trees were irrigated to replace their irrigation needs (INs) fully since spring 2018 when the crops were planted. INs were calculated as IN=*ET*_c_–*P*_e_, with *ET*_c_ being the maximum potential crop evapotranspiration calculated with the crop coefficient approach (Allen *et al.*, 1998) and *P*_e_ the effective precipitation recorded in the orchard (Orgaz and Fereres, 2001). The drip irrigation system installed consisted of a pipe along each row of trees with two drippers (4 litres h^–2^ each) 30 cm away from the base of the tree. Fertilization was applied following a carefully designed irrigation schedule.

### Meteorological variables

Meteorological variables were recorded using a weather station (Campbell Scientific Ltd, Shepshed, UK) located in the middle of the experimental area, with the meteorological sensors located at 3.5 m. The station recorded 30 min averages of air temperature (*T*_air_) and relative humidity, thus allowing the calculation of *D*Buck, 1981). In most cases, leaf temperature values from IRGA measurements were used to calculate the leaf-to-air *D*, which was a more precise estimation of the vapour pressure deficit driving leaf transpiration. The calculated *D* using leaf temperature is that shown in all figures, except in Supplementary Fig. S2, where the atmospheric temperature was used to indicate a whole *D* daily course. Solar radiation (*R*_s_) and wind speed (*u*) were also measured at the same station.

### Sap flux density

To obtain *J*_s_ (mm h^−1^), we monitored four central trees per species using the Compensation Heat Pulse (CHP) method (Green *et al.*, 2003), utilizing the Calibrated Average Gradient described in Testi and Villalobos (2009) for low values. Probe sets (IAS-CHP-AG, Cordoba, Spain) were installed on the south-facing side of the trunk, at 55 cm height on average, and *J*_s_ was measured at 5 mm depth in the xylem with heat pulses released every 30 min during the experimental period controlled by a CR6 datalogger connected to an AM25T multiplexer (Campbell Scientific Ltd). To determine the sensitivity of *J*_s_ to *D*, we plotted the data every 30 min, from dawn to mid-day on the days when *g*_s_ was measured. Several equations were tested, including logarithmic and parabolic equations (Supplementary Table S1). Parabolic equations showed the best fit (i.e. the highest *R*^2^) and they were used to calculate maximum sap flux density (*J*_smax_) and the corresponding *D* value at which *J*_smax_ occurred (*D*_*J*smax_) for all trees, using the procedure described in Grossiord et al. (2017, 2018). In brief, *J*_smax_ was extracted for each individual tree from the fitted parabolic relationships:


$$\begin{array}{l} J_{s}=a\times D^{2}+b\times D \end{array}$$ (1)

by calculating the location of the vertex (*h*) which in our case is *D*_*J*smax_:


$$\begin{array}{l} h=-b/2a \end{array}$$ (2)

and inserting it back to the Equation 1. In addition, we also modelled *J*_s_ as a linear function of the logarithm of *D*, with the slope $m_{J_{s}}\left\{cm\;h^{-1}{\left[ln\left(kPa\right)\right]}^{-1}\right\}$ representing the sensitivity of *J*_s_ to *D*, and the intercept *J*_sref_ representing transpiration at *D*=1 kPa, as proposed in Litvak *et al.* (2012) based on Oren *et al.* (1999):


$$\begin{array}{l} J_{s}=J_{sref}+m_{J_{s}}\times ln\left(D\right) \end{array}$$ (3)

### Stomatal conductance

Diurnal *g*_s_ was measured on three clear days from May to October for each species (21 May 21, 9 July, and 8 October for lemon, orange, and grapefruit, and 10 June, 23 July, and 28 September for almond and olive), every 30–60 min from dawn to dusk. However, in the Results, the data used were from dawn to mid-day in order to avoid hysteresis (Hernandez-Santana *et al.*, 2016), in two sun-exposed current-year leaves per instrumented tree. Three portable photosynthesis systems (Li-Cor 6400-XT, LI-COR, Lincoln, NE, USA) were used, equipped with a 2 × 3 cm standard chamber, at ambient light and CO_2_ conditions. Maximum stomatal conductance (hereafter written as measured *g*_smax_ to distinguish it from theoretical *g*_smax_) per individual and species was determined, the latter being obtained from averaging the maximum values of the four trees per species studied on every measurement day, which did not occur necessarily at the same time. Then, the *g*_s_ reduction was calculated by dividing the *g*_s_ at *D*_*J*smax_ by measured *g*_smax_. The *J*_smax_/*g*_smax_ ratio was calculated for each instrumented tree, as explained above. *J*_smax_ and measured *g*_smax_ were derived from the data collected during the days when *g*_s_ was measured, but they did not necessarily occur at the same time on the same day.

The approach explained in full detail in Hernandez-Santana *et al.* (2016) and extended on in Hernandez-Santana *et al.* (2018) was used to establish the relationships between *g*_s_ and the ratio of *J*_s_ to *D* (*g*_s_–*J*_s_/*D*) for each instrumented tree. The *J*_s_/*D* versus *g*_s_ calibration equations were established using the data of the three days when *g*_s_ was measured from dawn to mid-day (*n*=23–25) with the above-described methodological approach. The slopes of the individual relationships of the first day of measurement were compared with *H*_v_ to test our last hypothesis, to avoid redundancy as the results of the different days were similar (Table 1; Supplementary Table S2), and *H*_v_ was estimated only once throughout the whole measurement period. This relationship is considered a simplification of the inverted formula of Penman–Monteith for canopies tightly coupled to the atmosphere.

**Table 1.** Summary of the coefficient of determination (*R*^2^) of the first day of measurement

	Tree 1	Tree 2	Tree 3	Tree 4
Almond	**0.93**	**0.90**	**0.95**	**0.96**
Olive	**0.75**	**0.95**	**0.95**	**0.97**
Lemon	**0.90**	**0.94**	**0.62**	**0.89**
Orange	**0.79**	**0.93**	0.02	**0.76**
Grapefruit	0.00	0.09	0.24	0.06

Numbers in bold indicate significant relationships.

### Leaf vein density

To determine leaf vein density, defined as the length of vein per unit leaf area (mm mm ^–2^), one fully developed, current-year and sun-exposed leaf from six border individuals per species was used. The major first vein was not considered. Samples were lightly sanded before being introduced for 7 d in solutions of between 8 M and 5 M NaOH, depending on the species. Olive leaves needed longer periods, in some cases up to 40 d. A sodium hypochlorite solution was used to eliminate any extra pigment from the leaves after the chemical cleaning. Images of cleared leaves stained with 1% safranin were captured using a stereo microscope (Motic SMZ-171-TLED, Hong Kong, China) (Supplementary Fig. S1). Images of ~10 mm^2^ were taken using the software Motic Images Plus 3.0. Then the software ImageJ2 FIJI version 2.35 was used to quantify vein length and image size.

### Theoretical maximum stomatal conductance

Theoretical maximum stomatal conductance (written as theoretical *g*_smax_ to distinguish it from measured *g*_smax_) was calculated based on stomata size and density according to Franks *et al.* (2009):


$$\begin{array}{l} Theoretical\;g_{smax}=\frac{d\alpha D\sqrt{2S}}{v\left(0.5+0.627\sqrt{\alpha }\right)} \end{array}$$ (4)

Where *d* is the diffusivity of water in air (m^2^ s^–1^), α is a fraction of stomatal size (*a*_max_/*S*), *a*_max_ is the mean maximum stomatal pore area (m^–2^), *S* is the stomatal size (µm^2^), *D* is the density of stomata (number mm^–2^), and *v* is the molar volume of the air (m^3^ mol^–1^).

To estimate the stomatal size and density, we used two leaves from six sampled shoots per species from border trees—one leaf for the adaxial leaf side and the other for the abaxial leaf side. A central area of ~7 cm² was randomly selected on the leaf, and an epidermal impression was obtained by applying nail polish, allowing it to dry, and using transparent double-sided adhesive tape to transfer the impression to a microscope slide. After preparing the slides, we searched for stomata on the adaxial side on several leaves of the studied species and, after verifying that there were no stomata, we obtained only the abaxial side.

Stomatal density [i.e. the number of stomata per unit epidermal area (no. of stomata mm^–2^)] was calculated for each species as the mean of six plots (one section per leaf) at ×40 magnification using a light microscope (Motic BA210; Motic Microscopy, Hong Kong, China) (Supplementary Fig. S2) randomly chosen in one of the leaves sampled. Then, we calculated stomatal size by measuring stomatal length per stomatal width (μm^2^) of 20 stomata per leaf using the software Motic Images Plus 3.0. Finally, we use the mean value.

### Theoretical specific hydraulic conductivity

Six small branches per species, of ~5 cm diameter, were collected for anatomical characterization and fixed in FAA (5% formaldehyde, 2.5% acetic acid, 50% ethanol). Sections 15 µm thick were cut using a cryostat (Leica CM 1950), stained with a mix of safranin and astra blue (Gärtner and Schweingruber, 2013), and mounted in DPX. Images (Supplementary Fig. S3) were taken with a light microscope (OLYMPUS BX61), and the area of all the vessels present in a transect of the branch was measured using Fiji (Schindelin *et al.*, 2012). The equivalent diameter of the circle was calculated from these areas, and the theoretical specific hydraulic conductivity (*k*_s_) was determined using the Hagen–Poiseuille equation (Tyree and Ewers, 1991):


$$\begin{array}{l} k_{s}=\left(\frac{\pi \rho \;}{128\eta A}\right)\overset{n}{\underset{i=1}{\sum }}d_{i}^{4} \end{array}$$ (5)

where ρ and η are the density and viscosity of water at 20 °C (998.2 kg m^–3^ and 1.002 × 10^–9^ MPa*s, respectively), *A* is the area of the transect, and *d* is the equivalent diameter of every vessel within the transect.

### Plant hydraulic conductance

Plant hydraulic conductance (*K*_plant_), calculated from the *g*_s_ measured at the same time of the day as when Ψ_min_ was determined (around 13.00 h GMT), *D*, and pre-dawn and minimum leaf water potentials (Ψ_pd_ and Ψ_min_) were measured three times (21 May, 9 July, and 8 October for lemon, orange, and grapefruit, and 10 June, 23 July, and 28 September for almond and olive) during the study period. Leaf water potential was measured with a Scholander-type pressure chamber (PMS Instrument Company, Albany, OR, USA). We sampled two leaves from current-year shoots of the four central trees per species. The equation used was:


$$\begin{array}{l} K_{plant}=\frac{g_{s}\times D}{{\;\Psi }_{pd}-{\;\Psi }_{min}} \end{array}$$ (6)

### Huber value calculation

To obtain the *H*_v_, defined as the ratio between sapwood allocation relative to leaf area, we used 6–9 branches for each species collected in July, with diameters from 6.2 mm to 20 mm. The branches were sampled from border trees, and the total leaf area and basal sapwood area were measured. The diameters were measured with a calliper, and the corresponding leaf area was measured with a portable leaf area meter (LI-3000C, Li-Cor). With these data, we established the relationships (Supplementary Table S3) to estimate the leaf area from the instrumented trees by measuring the diameters of the highest order branches. Almost all the branches were in the range of the established relationships. However, in some cases, we extrapolated the results for wider branches. The diameter measurements of the instrumented trees were conducted from mid-August to September.

### Theroretical considerations of *H*_v_ for the mechanistic modelling of stomatal conductance using *J*_s_ measurements

The *H*_v_ is a hydraulic trait defined as the ratio of xylem area over leaf area (cm^2^ cm^–2^). It has been used to convert xylem area-specific conductivity (*K*_s_) into a more physiologically meaningful variable, such as leaf area specific conductivity *K*_L_ (*K*_L_=*K*_s_*H*_v_), thereby linking the unit-area water flux through plants with the water potential gradient necessary to drive that flux (Mencuccini *et al.*, 2019). Based on these considerations, we develop by analogy a trait-based predictive model for *g*_s_, linking the water loss through the leaf stomata with the xylem water flow using *H*_v_. As a starting point, we used the simplified calculation of *g*_s_ from transpiration measurements. This is based on the inversion of the simplification of the Penman–Monteith equation under certain circumstances, such as in tightly coupled canopies. In these conditions, transpiration is mainly regulated by stomatal conductance, depending on atmospheric conditions (McNaughton and Jarvis, 1983). Thus, we can calculate *g*_s_ (which can be expressed per leaf area as cm^3^ cm^–2^ s^–1^) from transpiration and atmospheric data as follows:


$$\begin{array}{l} g_{s}=\frac{\lambda \;\gamma \;E}{\rho C_{p}D} \end{array}$$ (7)

where *E* is tree transpiration, ρ is the air density, *C*_p_ is the heat capacity of air, *D* is the air vapour pressure deficit, λ is the latent heat of vaporization of water, and γ is the psychometric constant. Thus, although λ, γ, and ρ are temperature dependent, for our purpose they can be considered constant together with *C*_p_, compared with *E* and *D* that are variables with a large range of change for the conditions of our study. Therefore, Equation 7 could be written as follows:


$$\begin{array}{l} g_{s}=C\frac{E}{D} \end{array}$$ (8)

Where *C* includes all the constant factors of Equation 7, namely. $\frac{\lambda \;\gamma \;\;}{\rho C_{p}}$. Moreover, transpiration values can be estimated from sap flow-related measurements, such as *J*_s_ (which can be expressed per sapwood area as cm^3^ cm^–2^ s^–1^). Specifically, *E* can be calculated as the product of multiplying *J*_s_, measured at a single point in the sapwood, by the area in which it is conducted (i.e. sapwood area). Likewise, if we want to refer *E* per unit of leaf area, as these are the units of *g*_s_, it must be divided by the leaf area. Therefore, to go from *J*_s_ to *E*, what we need to do is to multiply *J*_s_ by *H*_v_:


$$\begin{array}{l} g_{s}=C\;H_{v}\frac{J_{s}}{D} \end{array}$$ (9)

The term *C* is the same for all the species. Therefore, Equation 9 will be used to test our hypothesis that *g*_s_ can be explained through *J*_s_ and the hydraulic trait *H*_v_. We expect that the slope of the relationship of *g*_s_–*J*_s_/*D* (unitless) is determined by the hydraulic trait *H*_v_, which can be considered species specific.

### Statistical analyses

We ran simple regression modelling to determine the relationships between *J*_s_/*D* and *g*_s_, *H*_v_ and the ratio *J*_smax_/*g*_smax_, *J*_s_ and *g*_s_ in response to *D*, and the slope of the previous relationship and *H*_v_. Differences between the species for the different variables considered (*D*_*J*smax_, $m_{J_{s}}$, reduction of *g*_s_, *H*_v_, *J*_smax_/*g*_smax_, calculated *g*_s_, measured *g*_s_, *K*_s_, leaf vein density, and *K*_plant_) were determined using linear models with Tukey’s post-hoc comparison. No random effect was used as only one measurement was made per tree, or, if multiple measurements were made, the values were averaged per tree. When no normal or heterocedastic residuals were obtained, an appropriate transformation of the variable was used, such as log transformation, square-root transformation, etc.

To further analyse the regulation of transpiration (represented by *J*_s_ as a proxy), a multitrait analysis such as a principal component analysis (PCA) was conducted using the function ‘prcomp’ from the Stats package and ‘fviz_pca_biplot’ from the factoextra package for its visualization (Kassambra and Mumdt, 2020). To model the influence of hydraulic traits on the level of relationships between *g*_s_ and *J*_s_/*D*, we fitted a regression equation. The *R*^2^ values of the same *g*_s_ and *J*_s_/*D* regression equations were used as the response variable and *K*_plant_ and *H*_v_ as predictor traits. For these analyses, we pooled together the data of all the studied species. Visual checks of model various assumptions (normality of residuals, normality of random effects, linear relationship, homogeneity of variance, multicollinearity) were conducted with ‘check_model’ from the performance package (Lüdecke *et al.*, 2021) to analyse the *R*^2^. Moreover, to calculate the relative importance metrics of the regressors of our models, we used the R-package relaimpo (Grömping, 2006). We calculated the relative importance in linear regression using the ‘lmg’ method in the ‘calc.relimp’ function (Grömping, 2006). Confidence intervals for relative importance metrics (‘booteval.relimp’) were calculated via bootstrapping (1000 runs) using the bootstrapping facility that the package provides (‘boot.relimp’). R software (R version 4.2.2) was used for all the analyses.

## Results

### Sap flux density response to vapour pressure deficit

**Fig. 1.**
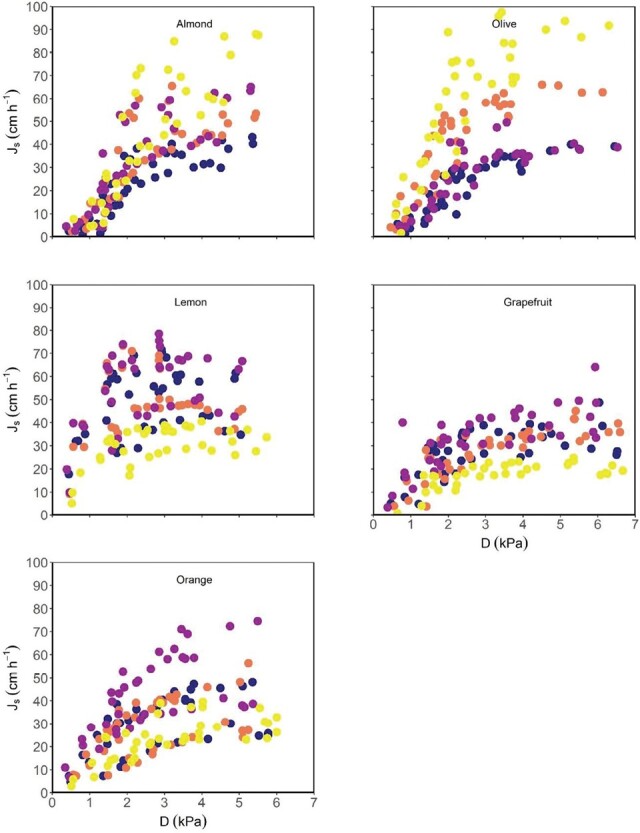
Response of sap flux density (*J*_s_) to vapour pressure deficit (*D*) for four almond, lemon, grapefruit, orange, and olive trees. Data were collected on three clear days from dawn to mid-day every 30 min. Different colours are different trees (*n*=4).

**Fig. 2.**
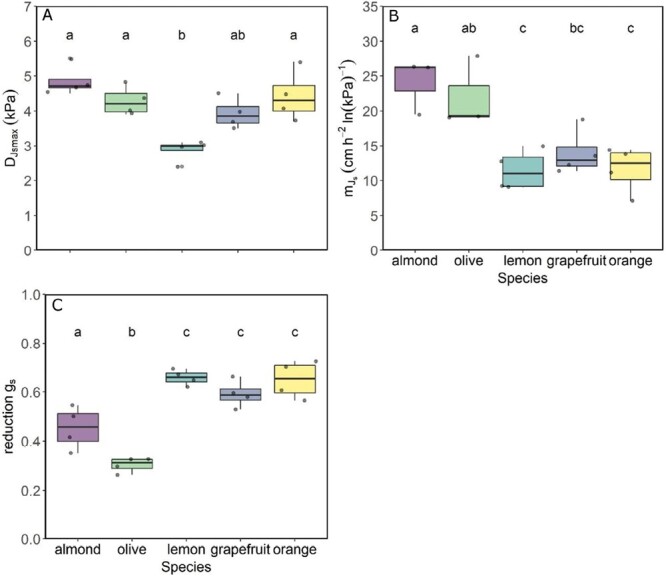
Boxplot of (A) the vapour pressure deficit at which maximum sap flux density occurs (*D*_*_J_*smax_), (B) sensitivity of sap flux density (*J*_s_) to vapour pressure deficit (*D*) ($m_{J_{s}}$=d*J*_s_/dln *D*), and (C) reduction of stomatal conductance (*g*_s_) at *D*_*_J_*smax_ compared with its maximum, calculated from data of the instrumented trees (*n*=4) over the three measurement days.

During the days when *g*_s_ was measured (Supplementary Fig. S4), *D* varied from ~0.1–0.2 kPa to almost 4.5 kPa (Supplementary Fig. S5). Half-hourly *J*_s_ from the monitored trees generally increased in response to increasing *D*. *J*_s_ varied from maxima of almost 100 cm h^–1^ in olive trees to 60 cm h^–1^ maxima in grapefruit individuals (Fig. 1). For low *D* values, *J*_s_ increased linearly with increasing *D*. However, as *D*_*J*smax_ was achieved, *J*_s_ either reached saturation or the slope became less steep for the increasing *D*, as in almond and olive, or even declined as in some *Citrus* trees, especially in lemon. Despite the intraspecific variability of *J*_s_ absolute values, the curve response to increasing *D* was very similar within trees of the same species and different among the species, pooling together the data from the three measurement days. Therefore, *D*_*J*smax_ was found to be dependent on the species, being significantly higher for almond, orange, and olive (~4.5–5 kPa on average) than for lemon, which was the lowest (~3 kPa) (Fig. 2A).

To analyse further the *J*_s_ limitation by *g*_s_, we assessed *D*_*J*smax_, $m_{J_{s}}$, and the reduction of *g*_s_ compared with measured *g*_smax_ at *D*_*J*smax_ among the studied species (Fig. 2). A coordinated pattern for the analysed variables was found. Almond, olive, and orange showed the greatest *D*_*J*smax_ (4.85 ± 0.22 kPa and 4.28 ± 0.21 kPa, for almond and olive, respectively), which indicates the least *J*_s_ limitation by *D*, and lemon had the lowest (2.88 ± 0.16 kPa), indicating the opposite. The highest response to *D*, $m_{J_{s}}$, was also shown by almond (significantly higher than the rest of the species) and olive (statistically similar to almond and grapefruit but significantly higher than the other two *Citrus* species). Almond and olive also showed a significantly lower reduction of *g*_s_ at *D*_*J*smax_ (0.45 ± 0.04 and 0.30 ± 0.02, for almond and olive, respectively) than the *Citrus* species. Although *Citrus* species did not show significant differences for the variables analysed, lemon had the most extreme behaviour.

### Coordination of anatomical and functional hydraulic traits with sap flux density regulation by species

**Fig. 3.**
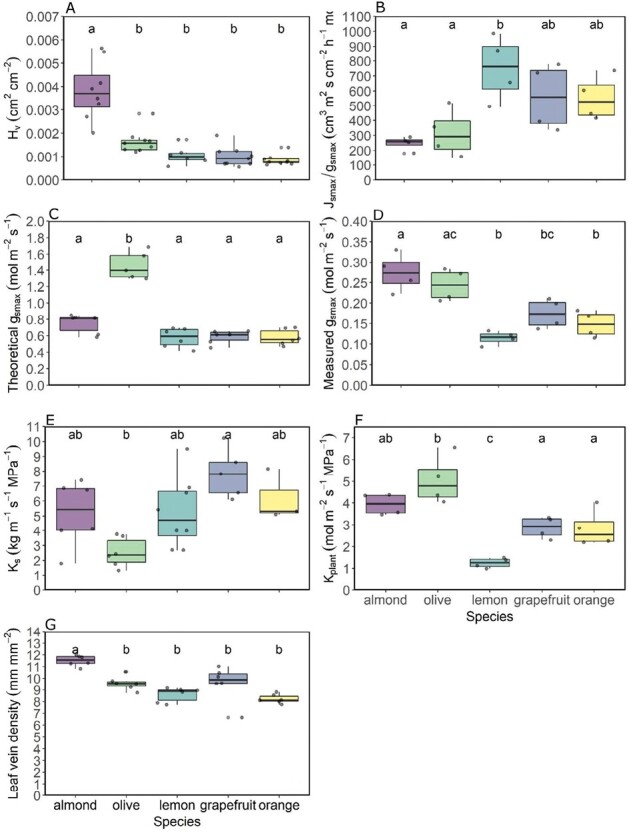
Boxplot of (A) the Huber value (*H*_v_), (B) the ratio between the measured maximum stomatal conductance (*g*_smax_) and the maximum sap flux density (*J*_smax_), (C) theoretical stomatal conductance (*g*_smax_) (D) measured maximum stomatal conductance, (E) calculated specific hydraulic conductivity (*K*_s_), (F) plant hydraulic conductance (*K*_plant_), and (G) leaf vein density by species. (A), (C), (E), and (G) have been calculated from anatomical samples of adjacent trees (*n*=6–9) and (B), (D), and (F) from data of the instrumented trees (*n*=4).

The functional hydraulic traits analysed (*J*_smax_/*g*_smax_, measured *g*_smax_, and *K*_plant_) showed an overall coordinated response across the species (Fig. 3). However, the ratio *J*_smax_/*g*_smax_ had the inverse pattern of the other two traits analysed. *J*_smax_/*g*_smax_ was smaller for almond and olive than for the *Citrus* species, *J*_smax_/*g*_smax_ being statistically lower for almond and olive than for lemon. When comparing measured *g*_smax_ with *K*_plant_, almond (0.27 ± 0.02 mol m^–2^ s^–1^ and 3.94 ± 0.24 mol m^–2^ s^–1^ MPa^–1^, measured *g*_smax_ and *K*_plant_, respectively) and olive (0.24 ± 0.02 mol m^–2^ s^–1^ and 5.04 ± 0.55 mol m^–2^ s^–1^ MPa^–1^, measured *g*_smax_ and *K*_plant_, respectively) had higher values than the *Citrus* species, with this difference being statistically significant with lemon (0.11 ± 0.01 mol m^–2^ s^–1^ and 1.25 ± 0.11 mol m^–2^ s^–1^ MPa^–1^, measured *g*_smax_ and *K*_plant_, respectively).

The coordination among the anatomically derived hydraulic traits was not as clear as with the former functional traits analysed. *H*_v_ had a very similar pattern to leaf vein density, with almond being significantly higher (11.51 mm mm^–2^ and 3.83 × 10^–3^ cm^2^ cm^–2^, for leaf vein density and *H*_v_, respectively) than for the rest of species. However, for the theoretical *g*_smax_, the highest value was shown by olive, which was significantly different from the rest. Almond presented the second highest theoretical *g*_smax_. In the case of *K*_s_, the only significant difference was between olive and grapefruit, with the latter having the highest *K*_s_.

These trends shown by the functional and anatomical hydraulic traits across the studied species, although more clearly for functional traits, were overall coordinated with those calculated with the *J*_s_ dataset (Fig. 2). Almond and olive showed the highest theoretical and measured *g*_smax_ as well as hydraulic capacity (Fig. 3) with the greatest $m_{J_{s}}$. In contrast, *Citrus* species, in general, were the species with the least theoretical and ­measured *g*_smax_, the most limited hydraulic system, and the smallest $m_{J_{s}}$ (Figs 2, 3).

**Fig. 4.**
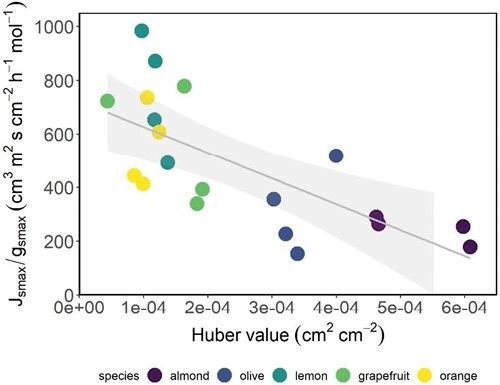
Relationship between the Huber value (sapwood area/leaf area) and the ratio between the measured maximum stomatal conductance (*g*_smax_) and the maximum sap flux density (*J*_smax_) monitored on the days when stomatal conductance was measured in the four instrumented trees per species.

The coordination analysis between functional and anatomical traits was performed visually and not quantitively because the functional and anatomical traits were measured in different individuals. Thus, a correlation performed with individual trees was not possible. Moreover, a correlation with species averages was not that useful as we had only five species, which is a low number for a correlation analysis. However, to further explore the relationship between anatomy and functionality integrating tree and leaf water transport levels, we compared the *H*_v_ with the ratio *J*_smax_/*g*_smax_, with the first being the anatomical and the second the functional trait at both species and individual level, because this is the only case where we had the measurements for the same trees. We applied the sapwood area–leaf area equation (Supplementary Table S3) to calculate the *H*_v_ of the instrumented trees where *J*_smax_ and *g*_smax_ were measured, allowing a direct comparison of individuals. The relationship between the *J*_smax_/*g*_smax_ ratio and *H*_v_ (Fig. 4) at the individual level showed a significant and negative linear relationship (*R*^2^=0.50, *P*<0.001), meaning that a higher sapwood allocation relative to leaf area produced a lower *J*_smax_/*g*_*s*max_. The highest *J*_smax_/*g*_smax_ and lowest *H*_v_ were shown by *Citrus* species and the lowest by almond. At species level, the linear relationship was not significant (*R*^2^=0.59, *P*=0.12) but the potential regression was statistically significant (*y*=5.83*x*^–0.661^, *R*^2^=0.78, *P*=0.04).

**Fig. 5.**
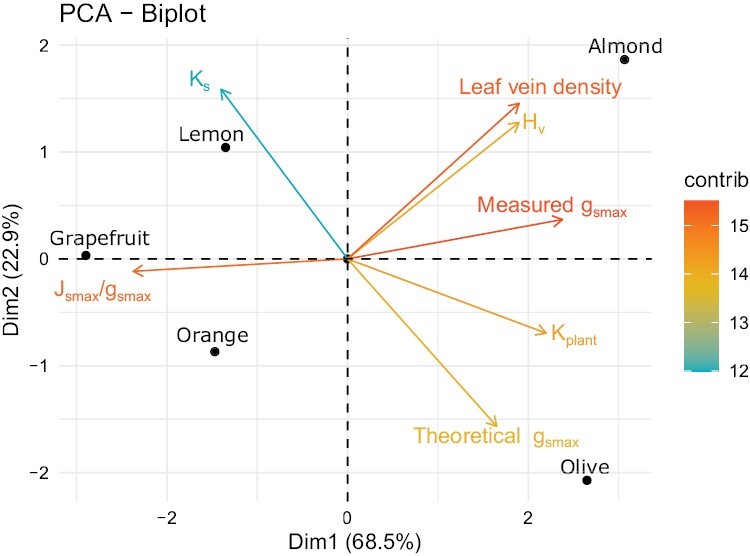
Principal component analysis representation with the hydraulic variables visualized in a gradient of colours depending on their contribution to the main dimensions analysed. Species are also added to visualize their relative positions on the plot. *H*_v_, Huber value; reduction *g*_s_, decrease of stomatal conductance compared with its maximum; *g*_smax_/*J*_smax_, the ratio between the measured maximum stomatal conductance and the maximum sap flux density; *K*_s_, calculated specific hydraulic conductivity; *K*_plant_, plant hydraulic conductance.

With the two main components obtained with the PCA (Fig. 5), we explained 95% of the variance of $m_{J_{s}}$. This result supports the coordination observed with the species by species analyses. The first component captures 70.6% of the variability of the hydraulic traits used, with the cumulative proportion of the first two being 91.6%. The first component seems to be related to the measured functional traits (*J*_smax_/*g*_smax_, reduction of *g*_s_, measured *g*_smax_, and *K*_plant_), while the second component is mainly related to the anatomically derived hydraulic traits (*H*_v_, theoretical *g*_smax_, leaf vein density, and *K*_s_). The variable contributing the least is *K*_s_. Adjusting the statistical model to explain $m_{J_{s}}$ with these two new variables obtained, we observed that the first one is significant and positive (*P*=0.012), with the model explaining up to 95% of the $m_{J_{s}}$ variability. Regarding the species results, it can be observed that *Citrus* species are located on the same side of component 1 while olive and almond are on the opposite side of the same component, corroborating the coordination patterns.

### Hydraulic traits-based modelling of the stomatal conductance–sap flux density relationship

**Fig. 6.**
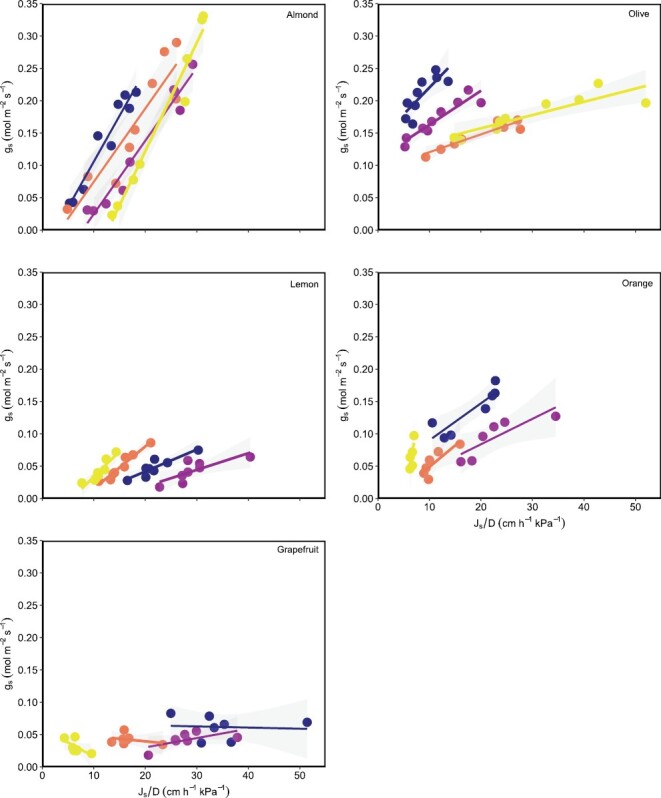
Relationship between the sap flux density/vapour pressure deficit ratio (*J*_s_/*D*) and stomatal conductance (*g*_s_) by species for the first day of measurement using data from dawn to mid-day measured in the four instrumented trees per species. Different colours are different trees.

**Fig. 7.**
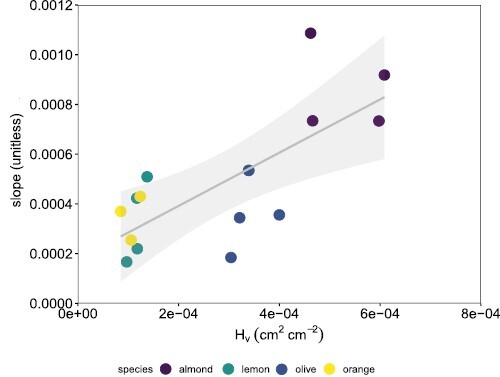
Regression between the Huber value (*H*_v_) and the slope of the relationships between stomatal conductance (*g*_s_) and the response of sap flux density (*J*_s_) to increasing vapour pressure deficit (*D*). Each point corresponds to one instrumented tree where the relationship *g*_s_–*J*_s_/*D* was statistically significant.

To continue exploring the impact of the studied species-specific hydraulic traits on the relationship between *g*_s_ and *J*_s_/*D* for the instrumented trees, we further calculated the determination coefficient of *g*_s_–*J*_s_/*D* relationships. The highest and significant $R_{g_{s-J_{s}/D}}^{2}$ values of the relationships were found for almond and olive (Fig. 6; Table 1; Supplementary Table S2), regardless of the day of measurement. Orange and grapefruit showed the lowest $R_{g_{s-J_{s}/D}}^{2}$, with some of those relationships being non-statistically significant. According to our mechanisitic hypothesis—that *g*_s_ can be explained through *J*_s_ and the hydraulic trait *H*_v_—the slope of those relationships was positively and linearly related to *H*_v_ (Fig. 7; *R*^2^=0.54, *P*=0.002).

Finally, we assessed the *H*_v_ effect on $R_{g_{s-J_{s}/D}}^{2}$ compared with the other major hydraulic trait measured in the instrumented trees: *K*_plant_. The fitted regression model between the$\;R_{g_{s-J_{s}/D}}^{2}$ and the predictors considered (*K*_plant_, *H*_v_) fulfilled various assumptions of the model (normality of residuals, normality of random effects, linear relationship, homogeneity of variance, and multicollinearity). The model accounted for $R_{g_{s-J_{s}/D}}^{2}$ variability of 47%, which was significant (*P*<0.01). The *H*_v_ was by far the major predictor selected, explaining 39% of the variability of the model (Table 2).

**Table 2.** Parameters of the models explaining the coefficient of determination of the tree relationships relating $\;R_{g_{s-J_{s}/D}}^{2}$ to hydraulic traits and their relative importance metrics with confidence intervals

	Intercept	*H*_v_	*K*_plant_	*R* ^2^
Model parameters	0.177	2547.143**	–0.05	0.47
Relative importance (%)		0.39	0.06	
Upper 95% CI		0.20	0.02	
Lower 95% CI		0.49	0.19	

All the species are included. **P<0.01.

## Discussion

### Relationship between sap flux density limitation by stomatal regulation with hydraulic traits

The field of plant hydraulics is a more recent focus within plant ecophysiology than traditional studies of plant–water relations (McCulloh *et al.*, 2019). Plant hydraulic traits represent the efficiency of water transport throughout the plant, and they are increasingly used in several disciplines, becoming a central hub integrating plant and ecosystem function (Sack *et al.*, 2016). However, their use in fields such as agricultural research is not that common despite their potential. What is even less abundant in the scientific literature is the evaluation of the coordination of hydraulic traits with the stomatal regulation of plant water use, employing multispecies measurements. In the few studies considering this approach, hydraulic traits were only measured at stem level (Litvak *et al.*, 2012; Grossiord *et al.*, 2017) or, as in the case of stomatal conductance, they were not measured but rather calculated (Flo *et al.*, 2021). Thus, our unique database allowed us to establish the role of different anatomical and functional hydraulic traits in the stomatal regulation of tree water use using agricultural trees with contrasting behaviours.

Under these premises, we found support for our main hypothesis of an overall coordination between the regulation of tree transpiration, by stomatal conductance as vapour pressure increases, and hydraulic traits. We observed this coordination with the interspecific comparisons and also with the modelling considering the five species as a whole. The greater overall hydraulic capacity found in almond and olive compared with the *Citrus* species, although not always statistically higher with the species by species analyses (Fig. 3), was also highlighted by the PCA (Fig. 5). Thus, the hydraulic characteristics allowed almond and olive to maintain tree transpiration at higher VPD values (i.e. *D*_*J*smax_) than *Citrus* species (Fig. 2). These characteristics would facilitate the transport of larger amounts of water to leaves for transpiration in almond and olive than in *Citrus* species, maintaining their stomata open. As a consequence, the higher stomatal conductance promotes higher CO_2_ assimilation rates, which favours growth.

According to our hypothesis, among all the hydraulic traits analysed, the most relevant was *H*_v_. This trait relates the area between sapwood and leaves, the tissues involved in water transport and consumption, and it has received a lot of attention lately as a major trait (Mencuccini *et al.*, 2019). Our data confirm that the species with the strongest stomatal control (*J*_s_ limitation at lower *D*, lower *g*_s_, etc.) are those with the lowest *H*_v_ (Fig. 3). We also found that *H*_v_ was significantly related to the slopes of the relationship between stomatal conductance and the response of tree transpiration to increasing VPD (Fig. 7) besides contributing the most in explaining the coefficient of determination of these same relationships (Table 2). The relationship *g*_s_–*J*_s_/*D* has been used successfully to monitor *g*_s_ in a continuous manner with *J*_s_ and *D* measurements in individuals of different species and ages, and with various objectives (Hernandez-Santana *et al*., 2016, 2018; Li *et al.*, 2022; Perez-Arcoiza *et al.*, 2022). Stomatal conductance is a key variable in plant water and carbon relations (Buckley, 2019) but, unfortunately, it cannot be measured continuously and automatically as there is no technology available for this. Therefore, the possibility of estimating *g*_s_ with relatively simple measurements of *J*_s_ and a meteorological measurement included in practically any weather station, such as *D*, can be of great use. Until now, the model to estimate *g*_s_ was empirical despite being based on robust physiological concepts. The inclusion of the *H*_v_ trait in the model means that it can be applied more mechanistically, which greatly simplifies its use. More work is needed to confirm this general trait-based model as we tested it using five species. Furthermore, the significant relationship between *H*_v_ with its novel functional proxy, *J*_smax_/g_smax_ (Fig. 4), showed direct experimental evidence of a relationship between tree water use, leaf gas exchange regulation, and a hydraulic trait. The significant relationship between *H*_v_ and *J*_smax_/*g*_smax_ also demonstrated that an anatomically calculated trait, such as *H*_v_, is related to a functional one, such as *J*_smax_/*g*_smax_. The *H*_v_–*J*_smax_/*g*_smax_ relationship was negative: almond, the species with the highest *H*_v_, showed the lowest *J*_smax_/*g*_smax_, and *Citrus* species had the opposite behaviour. The relationship between *H*_v_ and *J*_smax_/*g*_smax_ suggests that a larger sapwood area would compensate for a lower *J*_s_ in relation to lower leaf area with higher stomatal conductance. This is an analogous explanation to the—also negative—relationship between *H*_v_ and xylem hydraulic conductivity found in other works (Choat *et al.*, 2011; Togashi *et al.*, 2015; Mencuccini *et al.*, 2019), where the low hydraulic conductivity is said to be compensated for by larger sapwood area to maintain rates of water transport. In other words, given that the amount of leaves supported by a particular area of sapwood is limited by the sapwood capacity to supply water, this limitation would be compensated by this *J*_smax_/*g*_smax_ ratio. Besides being significantly related to the *J*_smax_/*g*_smax_ ratio, in our study, average *H*_v_ by species was also significantly and positively related to leaf vein density (*R*^2^=0.87, *P*=0.02). This relationship can be interpreted such that species that invest more in vascular tissue in stems in relation to leaf area also invest more in vascular tissue in leaves. Given that a higher *H*_v_ means a larger sapwood area for a smaller leaf area, leaf vein density would increase to maintain water transport through the plant to the atmosphere. The rest of the relationships between other hydraulic traits and *H*_v_ showed trends, but they were not significant likely due to the fact that only averages per species (*n*=5) were used as *H*_v_, and the rest of hydraulic traits, were sampled in different trees (see the Materials and methods). These relationships are beyond the scope of this work, and they are not shown. However, these findings add to the increasing body of recent evidence recognizing the importance of *H*_v_ in plant water balance and drought responses studies (Mencuccini *et al.*, 2019; Rosas *et al.*, 2019; Xu *et al.*, 2021), whose coordination with key plant functional traits is still poorly understood.

### Coordination between anatomical hydraulic traits with their function

In contrast to the above-mentioned *H*_v_–*J*_smax_/*g*_smax_ negative relationship (Fig. 4), the rest of the anatomical hydraulic traits studied showed an overall positive coordination with their related functional traits (Fig. 3). However, the statistical differences among the species were not always the same across the studied variables.

We observed that leaf vein density was overall coordinated with *K*_plant_. Leaf vein density would play a major role in the whole-plant hydraulics, as water transport in the leaves has been found to constitute up to 70% (Sack and Holbrook, 2006) of the whole-plant resistance to water flow despite representing only a very small fraction of the whole-plant transport distance (Brodribb *et al.*, 2010). Although we did not measure leaf hydraulic resistance, we used the leaf vein density as a proxy for this variable (Sack and Frole, 2006; Brodribb *et al.*, 2007; Scoffoni *et al.*, 2011) because a greater leaf vein density indicates a greater efficiency in transporting water through the leaf (Sack and Frole, 2006; Brodribb *et al.*, 2007). Despite the general coordination, there were some differences between *K*_plant_ and leaf vein density, which could be explained by the leaf hydraulic resistance not being the major resistance of the plant for all the species studied, and thus not the major limitation (Sack and Holbrook, 2006). Recent studies have pointed to below-ground components, such as soil–root hydraulic resistance, to greatly contribute to the whole-plant hydraulic resistance due to the disconnection, among other events, at the soil–root interface level (Rodriguez-Dominguez and Brodribb, 2020; Bourbia *et al.*, 2021; Duddek *et al.*, 2022).

However, there are other explanations for the differences across the species between the anatomical trait leaf vein density and a similar functional trait such as *K*_plant_, involving non-anatomical processes that are not included in the measurement of leaf vein density but that influence *K*_plant_. Indeed, aquaporins could also be contributing to the limitation of the hydraulic conductance (Kaldenhoff *et al.*, 2008; Shatil-Cohen, 2011), but they are not reflected in leaf vein density. Another explanation is related to the degree of loss of xylem conductivity when maximum transpiration occurs. We assumed that stomatal conductance regulates tree transpiration to prevent any negative effect on the plant hydraulic conductance (i.e. cavitation onset; Martin-StPaul *et al.*, 2017; Creek *et al.*, 2019; Corso *et al.*, 2020). Because there was no soil water deficit, the regulation of stomatal conductance in response to increasing *D* could have been enough to reduce the risk of plants to reach water potential values inducing runaway embolism formation (Martin-StPaul *et al.*, 2017) to avoid any loss of plant hydraulic conductance. However, Manzoni *et al.* (2013) proposed an alternative explanation showing that some plant species regulate transpiration through stomata to function near maximum transpiration, allowing some loss of xylem conductivity, probably at peripheral levels, in well-watered conditions. According to these authors, there is an optimum balance between the driving force and cavitation occurring at intermediate water potentials, which defines the maximum transpiration rate that the xylem can sustain. In that sense, leaf vein density would be closer to the maximum hydraulic capacity to transport water than *K*_plant_, which could reflect some degree of cavitation. However, recent studies showed that some degree of cavitation occurring when ­stomatal closure takes place would provide some negative stomatal safety margins (Skelton *et al.*, 2020), which are uncommon for the environmental conditions of the studied area so the alternative explanation of Manzoni *et al.* (2013) would not explain the differences between *K*_plant_ and leaf vein density.

A similar rationale, whereby the non-inclusion of non-anatomical processes in anatomical traits would explain the differences between these and comparable functional traits, would also help to understand the differences between the rest of the anatomical–functional trait comparisons. This is the case for *K*_s_ which presents the most remarkable differences between the calculated, anatomical traits with its physiological trait, *K*_plant_. Calculation of *K*_s_ would need to incorporate, for example, pit membrane resistances for a better approximation of actual *K*_s_ (Jansen *et al.*, 2011; Lens *et al.*, 2011), more comparable with *K*_plant_. Moreover, according to our former reasoning that *K*_leaf_ could make up to 70% of *K*_plant_, *K*_s_ must not be that important. The rationale to explain the differences between anatomical traits and their functional analogues, namely anatomical traits not incorporating non-anatomical key processes, would also explain the much higher values of theoretical *g*_smax_ compared with measured *g*_smax_. It has been reported that the effective area of the stomatal pore could be smaller than the anatomical maximum considered in the theoretical *g*_smax_ calculation (Sack and Buckley, 2016). Murray *et al.* (2020) have quantified this relationship between operational or measured stomatal conductance and theoretical or calculated stomatal conductance across biomes, growth habits, and habitats, as the ratio measured *g*_smax_:theoretical *g*_smax_, and found a consistent value of 0.26 ± 0.11 (mean ±SD). This is consistent with our results where the ratio is 0.26 ± 0.07, with olive being the species with the ratio furthest from average at 0.18. The fact that the calculations of anatomical features do not include certain characteristics explains in particular why in olive the difference between the theoretical *g*_smax_ and the measured *g*_smax_ was even greater than for the rest of these species. Indeed, the presence of leaf surface features such as hairs surrounding the stomata, not included in the formula to calculate theoretical *g*_smax_ anatomically, may have limited the diffusion through stomata in olive (Bickford, 2016), limiting evaporation, and thus explaining the greater difference in olive than in the rest of the species since it is the only species which has trichomes. Thus, although anatomically calculated hydraulic traits are interesting for comparing species because they follow a general trend similar to the functional traits, there may be important exceptions because the anatomical traits do not include key information.

### Implications and future directions

Our multispecies database combining a proxy of water use, *J*_s_, and gas exchange with several hydraulic traits allowed us to conduct a hydraulic trait-based approach, both at the individual level comparing experimental data by species and by modelling considering the five studied species as a whole. Our results contribute to the identification of the key characteristics determining the causal relationship between water consumption and its regulation, which remain largely unexplored (Flo *et al.*, 2021). In fact, we are unaware of an explicit study evaluating the effect of hydraulic traits on the regulation of tree water use by stomatal conductance across different co-existing species. Specifically, the trait-based *g*_s_ model helps to monitor *g*_s_ in an automatic and continuous manner. In turn, the *g*_s_ monitoring in relation to hydraulic traits can contribute to improve simulations and predictions of global vegetation and ecosystem models through the more mechanistic use of coordination of hydraulic traits with gas exchange instead of the empirical function representing plant water limitation used at present.

The predominant role of *H*_v_ in the relationship between water transpiration and its regulation by increasing *D*, either by its influence on the modelling of this relationship (Table 2; Fig. 7) or by direct comparison with its ecophysiological counterpart (*J*_smax_/*g*_smax_) (Fig. 4), adds more evidence to the key role played by *H*_v_ in water relations, which deserves further investigation. Related to the former, the link between anatomical traits, more easily measured with their functional process, could be of great help for phenotyping (Stahl *et al.*, 2020), although their use must be carefully considered. The observed difficulties in the comparison between anatomically derived hydraulic traits and functional traits may help to build new approaches for functionally correcting the calculated anatomic traits.

The interaction between atmospheric and soil drought deserves further exploration, as does the use of other hydraulic traits more related to the vulnerability of the system, in order to have a more complete vision of the studied coordination. The semi-arid climate of the studied area provided an extremely large range of *D* (almost 4.5 kPa on the days when the study was conducted), which allowed us to study the stomatal control of tree transpiration in response to that increment in atmospheric drought, presumably to protect the plant hydraulic system. However, the lack of soil water deficit and hydraulic vulnerability measurements did not allow us to fully study the species response to drought conditions combining both atmospheric and soil drought, which may increase the tension in the xylem. Under these conditions, cavitation episodes within the plant vascular system may appear (Tyree and Zimmermann, 2002), despite stomatal regulation, increasing the complexity of the implications of our study and deserving further exploration.

The challenge of maintaining crop production under climate change (Schauberger *et al.*, 2017) remains since the physiological traits have been barely evaluated in crops, compared with stomatal conductance and photosynthesis (Gleason *et al.*, 2017). To date, the development of drought-resistant or high productive cultivars has primarily relied on progress in the selection of morpho-anatomical traits related to drought resistance and improved water use efficiency (i.e. the ratio between the amount of water transpired and the biomass produced by a crop) using genomics and genetic tools. Yet, an essential aspect of increasing drought resistance in crops is to identify key hydraulic traits that ensure xylem safety under drought at the same time that high hydraulic efficiency is maintained, and to ensure that their impacts on leaf gas exchange regulation and growth are understood. Our results contribute to filling the existing gap in knowledge on these relationships in woody tree crops that can be used in future breeding programmes.

### Conclusions

We conclude that there is an interspecific relationship between stomatal regulation of transpiration and the hydraulic traits considering the five species studied. Among those we have studied, the trait that emerges with a more predominant role is *H*_v_, relating tree water use and stomatal conductance, adding to the growing body of evidence revealing a key role for *H*_v_ in plant water and carbon relationships.

There was an overall coordination between anatomical and functional hydraulic traits for the five species. However, we conclude that the anatomical traits do not include all the factors conditioning their function, and thus some differences arose when statistically comparing the behaviour of the species at the anatomical and functional level, which opens up new avenues for correcting anatomically derived traits. Although anatomical hydraulic traits could be very useful, our conclusion is that they should be used with caution.

Nevertheless, this study provides novel insights into the hydraulic basis for the regulation of tree water use across five tree crop species with relevant implications for phenotyping and in particular for *g*_s_ functioning and modelling.

### Supplementary data

The following supplementary data are available at *JXB* online.

Fig. S1. Leaf vein images.

Fig. S2. Stomata images.

Fig. S3. Xylem images.

Fig. S4. Daily variation of stomatal conductance measured during three different days.

Fig. S5. Daily course of vapour pressure deficit, solar radiation, and wind speed for the measurement days for the different species.

Table S1. Logarithmic and parabolic equations fitted to the vapour pressure deficit and sap flux density dataset.

Table S2. Summary of the coefficient of determination (*R*^2^) of the second and third day of measurement.

Table S3. Relationship between branch diameter (mm) and leaf area (cm^2^).

## Supplementary Material

erad157_suppl_Supplementary_Tables

erad157_suppl_Supplementary_Figures

## Data Availability

The data supporting the findings of this study are available from the corresponding author, Virginia Hernandez-Santana, upon request.
