# Supplementary material for: Role of hydraulic traits in stomatal regulation of transpiration under different vapour pressure deficits across five Mediterranean tree crops
Source: J Exp Bot. 2023 Apr 28;74(15):4597–612. doi: 10.1093/jxb/erad157 (PMC10433928; doi:10.1093/jxb/erad157)
Supplement: erad157_suppl_Supplementary_Tables [file erad157_suppl_supplementary_tables.pdf]

Table S1.

Species	Tree	Equation	R^2
Almond	1	y = 19.508ln(x) + 8.6485	0.78
	2	y = 26.188ln(x) + 12.545	0.70
	3	y = 26.361ln(x) + 15.143	0.73
	4	y = 42.257ln(x) + 9.3367	0.75
	1	$y = -2.263x^2 + 21.279x - 11.562$	0.80
	2	$y = -3.7457x^2 + 32.277x - 18.184$	0.73
	3	$y = -2.6133x^2 + 26.698x - 10.737$	0.74
	4	$y = -2.9569x^2 + 34.743x - 21.374$	0.76
Olive	1	y = 19.065ln(x) + 7.1352	0.85
	2	y = 27.852ln(x) + 21.019	0.84
	3	y = 19.276ln(x) + 10.501	0.70
	4	y = 40.261ln(x) + 27.057	0.78
	1	$y = -1.5471x^2 + 17.207x - 8.8612$	0.87
	2	$y = -3.1519x^2 + 30.526x - 8.7305$	0.85
	3	$y = -2.2386x^2 + 21.282x - 9.9587$	0.74
	4	$y = -4.1427x^2 + 41.412x - 12.55$	0.79
Lemon	1	y = 12.76ln(x) + 37.65	0.28
	2	y = 9.0399ln(x) + 40.625	0.17
	3	y = 15.015ln(x) + 42.504	0.34
	4	y = 9.2018ln(x) + 21.882	0.45
	1	y = -4.094x2 + 26.642x + 12.666	0.37
	2	y = -4.7288x2 + 27.555x + 15.641	0.38
	3	$y = -4.2462x^2 + 28.605x + 15.558$	0.41
	4	y = -2.0669x2 + 15.601x + 6.3805	0.54
Grapefruit	1	y = 12.268ln(x) + 18.063	0.39
	2	y = 13.522ln(x) + 16.557	0.38
	3	y = 18.8ln(x) + 26.898	0.52
	4	y = 11.333ln(x) + 12.306	0.58
	1	y = -2.3101x2 + 18.898x - 1.2186	0.46
	2	y = -1.7892x2 + 15.951x + 1.2343	0.38
	3	y = -3.2857x2 + 27.05x - 0.3701	0.56
	4	y = -1.3989x2 + 13.399x - 1.33	0.61
Orange	1	y = 11.139ln(x) + 17.144	0.51
	2	y = 14.349ln(x) + 13.293	0.76
	3	y = 13.795ln(x) + 21.146	0.63
	4	y = 7.0445ln(x) + 9.6922	0.59
	1	y = -1.5613x2 + 14.536x + 2.2678	0.56
	2	y = -1.0661x2 + 12.567x + 1.524	0.76
	3	y = -1.2419x2 + 13.496x + 7.7658	0.62
	4	y = -0.9566x2 + 9.3111x - 0.3366	0.68

Table S2.

	Day 2				Day 3			
	Tree 1	Tree 2	Tree 3	Tree 4	Tree 1	Tree 2	Tree 3	Tree 4
**ALMOND**	**0.75**	**0.63**	**0.80**	**0.78**	**0.69**	**0.96**	0.13	**0.59**
**OLIVE**	**0.69**	**0.72**	**0.56**	**0.89**	**0.53**	**0.57**	**0.88**	0.05
**LEMON**	0.18	**0.47**	0.16	0.24	**0.84**	**0.78**	**0.83**	**0.75**
**ORANGE**	0.28	0.00	0.60	0.01	0.08	0.01	0.64	0.38
**GRAPEFRUIT**	0.00	**0.43**	0.02	0.02	0.12	0.09	**0.71**	0.08

Table S3.

**Species**	**Equation**	**R^2**	**P**	**N branches**
almond	$y = 60.507e^{0.2448x}$	0.60	<0.05	8
olive	$y = 257.89e^{0.1922x}$	0.93	<0.0001	9
lemon	$y = 84.077e^{0.3844x}$	0.83	<0.05	6
grapefruit	$y = 214.22e^{0.2655x}$	0.75	<0.01	9
orange	$y = 935.42e^{0.1345x}$	0.66	<0.05	9
