# Supplementary material for: Role of hydraulic traits in stomatal regulation of transpiration under different vapour pressure deficits across five Mediterranean tree crops
Source: J Exp Bot. 2023 Apr 28;74(15):4597–612. doi: 10.1093/jxb/erad157 (PMC10433928; doi:10.1093/jxb/erad157)
Supplement: erad157_suppl_Supplementary_Figures [file erad157_suppl_supplementary_figures.pdf]

Figure S1. Leaf vein pictures

Figure S2. Stomata pictures.

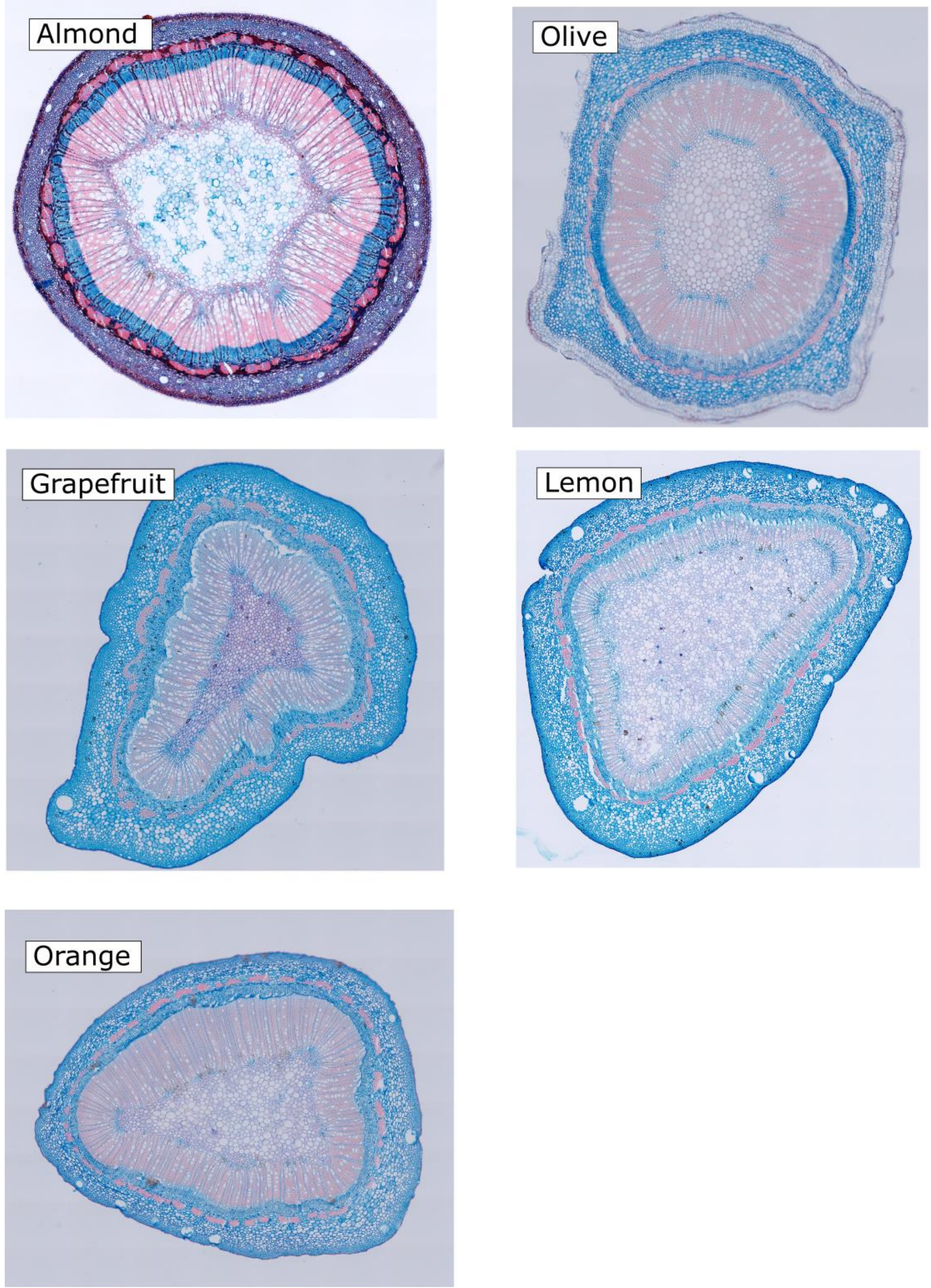

Figure S3. Xylem pictures.

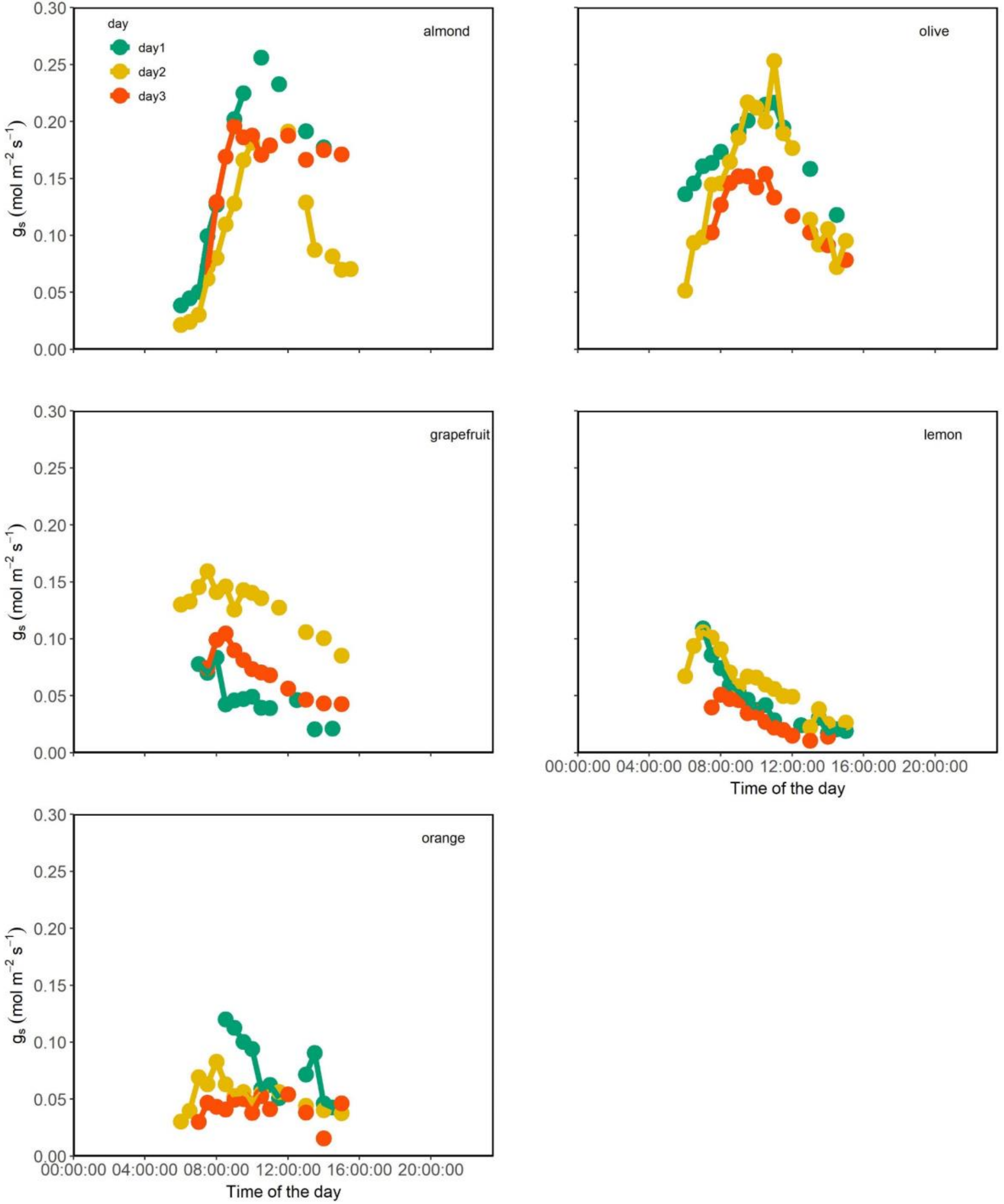

Figure S4. Daily variation of stomatal conductance measured during three different days. Each point represents the average of two leaves per four individuals per species (n=8).

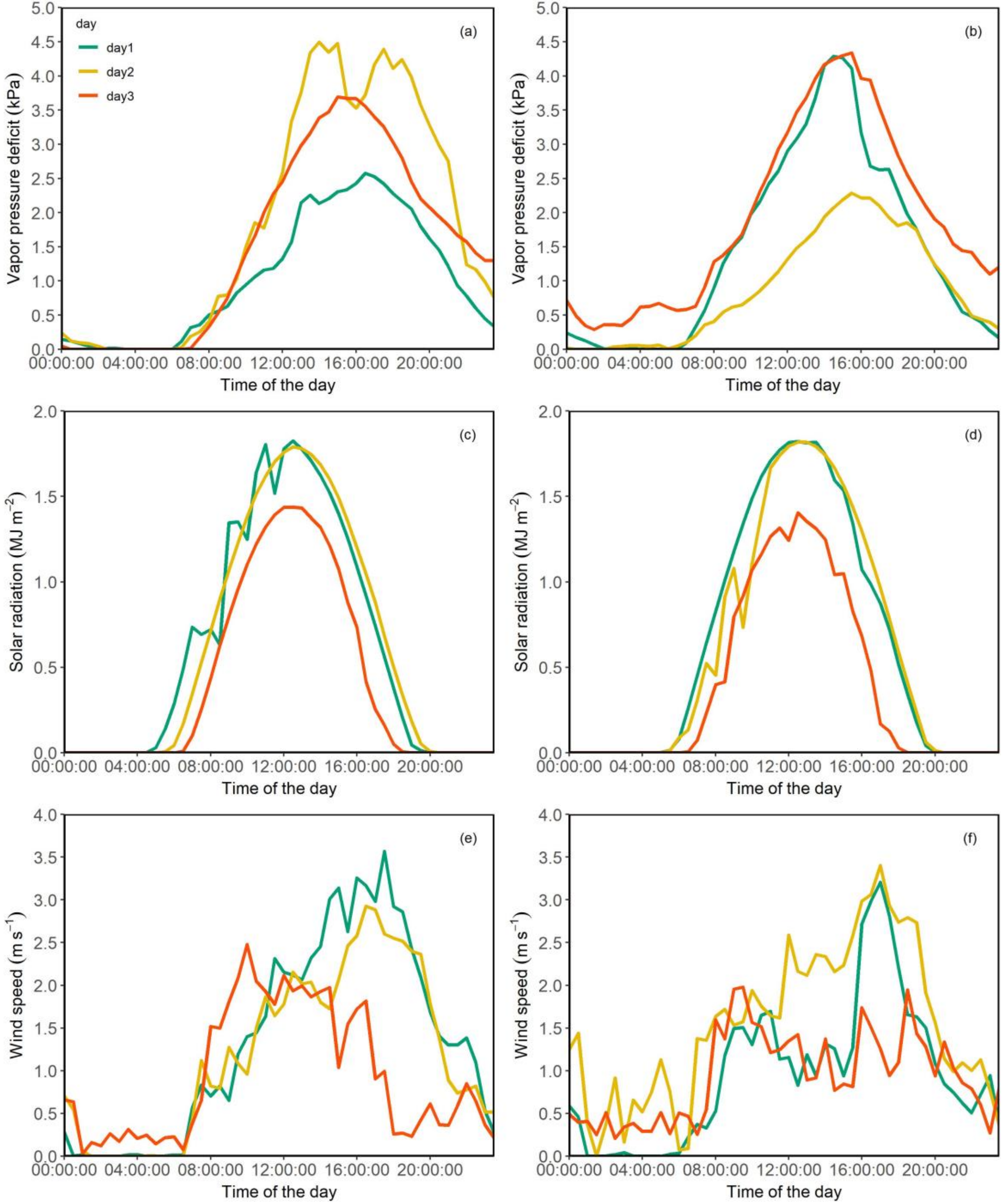

Figure 5. Daily course of vapor pressure deficit, solar radiation and wind speed for the three measurement days for olive and almond (a, c and e) and for grapefruit, lemon and orange (b, d and f).
